# From Treatise to Test: Evaluating Traditional Remedies for Anti-Biofilm Potential

**DOI:** 10.3389/fphar.2020.566334

**Published:** 2020-10-28

**Authors:** Snehal Kadam, Vandana Madhusoodhanan, Anuradha Bandgar, Karishma S. Kaushik

**Affiliations:** ^1^ Institute of Bioinformatics and Biotechnology, Savitribai Phule Pune University, Pune, India; ^2^ Dr. Anuradha’s Ayurvedic Clinic, Pune, India

**Keywords:** biofilm, treatise, antimicrobial, wounds, ayurveda

## Abstract

Traditional plant-based remedies hold vast potential as novel antimicrobial agents, particularly for recalcitrant infection states such as biofilms. To explore their potential, it is important to bring these remedies out of historical treatises, and into present-day scientific evaluation. Using an example of Indian traditional medicine (Ayurveda), we present a perspective toward evaluating historical remedies for anti-biofilm potential. Across compendia, we identified three plant-based remedies (of *Kalanchoe pinnata*, *Cynodon dactylon*, and *Ocimum tenuiflorum*) recommended for wounds. The remedies were reconstituted in accordance with historical practices, and tested for their effects on biofilm formation and eradication assays of wound pathogens, *Pseudomonas aeruginosa* and *Staphylococcus aureus*. Based on our approach and the results obtained, we provide insights into the considerations and challenges related to identifying potential remedies in historical texts, and testing them in the laboratory with standard biofilm assays. We believe this will be relevant for future studies exploring anti-biofilm approaches at the interface of historical medicine and present-day scientific practices.

## Introduction

Biofilms are multicellular aggregates of bacteria, attached to each other or a surface, via a self-produced extracellular matrix (Flemming et al., 2016). Implicated in a range of infection states from non-healing wounds to life-threatening burn-related sepsis (Hall-Stoodley et al., 2004; Kennedy et al., 2010; Chen and Wen, 2011; Høiby et al., 2011), biofilms are notoriously tolerant to conventional antibiotics, resulting in persistent infections and limited therapeutic options (Høiby et al., 2010; Sun et al., 2013; Ciofu et al., 2017; Sharma et al., 2019). Consequently, there is a concerted push to develop novel anti-biofilm agents, that will expand therapeutic options, and prolong the lifetime of current antibiotics. Traditional medical practices have been recognized as valuable sources of potential new antimicrobial agents (Yuan et al., 2016). These practices employ natural products, often from plant sources, thereby providing a huge diversity of active ingredients and biological targets. Further, traditional remedies are formulated using rudimentary processes that retain the crude and combined nature of the ingredients, which is possibly important for antimicrobial activity. As evident, principles that guide historical medical approaches differ from that of conventional antibiotics (Leekha et al., 2011). Therefore, prospecting historical antimicrobial remedies as potential anti-biofilm agents could provide a paradigm shift in the treatment of biofilms.

For this, it is essential to bring traditional medicinal remedies out of esoteric medical treatises and into scientific manuscripts (Harrison et al., 2015; Fuchs et al., 2018; Anonye et al., 2020; Furner-Pardoe et al., 2020). To fully explore this, it is imperative to enable the steady identification and evaluation of traditional formulations with anti-biofilm potential. In the context of traditional medical practices, this starts with probing historical treatises for potential remedies and testing them for anti-biofilm activity (“treatise to test”), following which promising agents can be taken up for further evaluation.

In this perspective article, we use Indian traditional medicine (Ayurveda) as an example, to search historic documents for evidence of biofilm-mediated infections, identify and reconstitute plant-based traditional remedies with anti-biofilm potential, and test them in the laboratory with standard biofilm assays. Based on gleanings across compendia, three plant-based remedies, of *Kalanchoe pinnata* (Lam.) Pers. (“*Parnabeeja*”), *Ocimum tenuiflorum* L. (Holy basil or “*Tulsi*”), and *Cynodon dactylon* (L.) Pers. (Bermuda grass or “*Durva*”) were reconstituted in sesame oil, and “whole” remedies were tested for their effects on biofilm formation and eradication of pre-formed biofilms using *in vitro* microtiter based biofilm assays. The three plant-based remedies showed varied inhibitory effects on biomass and metabolic activity of *P. aeruginosa* and *S. aureus* biofilms, with sesame oil alone also showing anti-biofilm effects. Notably, comparison of the effects on biofilm and planktonic growth states revealed differential effects on the two modes of growth. We discuss our approach and results, and leverage them to provide insights into the considerations and challenges relevant to evaluating traditional remedies as anti-biofilm agents.

## Methods

### Preparation of Plant-Based Whole Remedies

The medicinal plants were obtained from a private garden in Pune, India (Latitude 18.591,934, Longitude 73.793,037) belonging to Dr. Anuradha. The entire procedure was carried out with freshly-picked plant parts (plants were directly made into a paste and not stored). The leaves of *Kalanchoe pinnata* and *Ocimum tenuiflorum*, or grass of *Cynodon dactylon* (no other plant parts such as roots, shoots, stem or flowers were used), were made into a paste using a mechanized grinder (Kenstar multi-processor, Model No: MF0204) till a thick paste was obtained. For every one part of plant paste (100 ml), 16 parts of distilled water (1,600 ml) and four parts of sesame oil (400 ml) were used, resulting in a 1:16:4 ratio (by volume) of plant, water, and oil respectively. The plant paste, water and oil were mixed together in a wide-mouthed steel vessel to allow for evaporation of water. The mixture was boiled under low heat with stirring till the water completely evaporated (5–6 h), leaving the extracts from the plant paste in the sesame oil. The extract-containing oils were filtered in a single-fold cotton cloth, to remove any remaining chunks of the leaf paste, and stored at room temperature. These remedies were prepared in a single batch, and all testing was performed with the same batch. This boiling procedure was also carried out for a control of sesame oil alone, where one part of plant paste in the original ratio was replaced with water. Whole-pressed plant specimens were deposited in the herbarium (indexed with NYBG Steere Herbarium) at the Department of Botany, Savitribai Phule Pune University (SPPU). The retention vouchers for the plant specimens are *Cynodon dactylon* (L.) Pers. SPPU 0125, *Kalanchoe pinnata* (Lam.) Pers. SPPU 0124 and *Ocimum tenuiflorum* L. SPPU 0123.

### Testing the Effects of Plant-Based Whole Remedies on Biofilm Formation

To evaluate effects on the formation of biofilms, reconstituted formulations were added to bacterial cultures of *P. aeruginosa* (PAO1-pUCP18 (Irie et al., 2012)) or *S. aureus* (Strain AH 133 (Malone et al., 2009)), which were then allowed to grow under static conditions. Briefly, overnight cultures of *P. aeruginosa* and *S. aureus* were diluted to a concentration of 10^7^ cells/ml in fresh LB broth (dilutions based on optical density measured at A_595_). From this diluted culture, 10 µl was added to a clear, U-bottom non-treated 96-well plate (Corning) to give a final count of 10^5^ cells in each well (in triplicates). Following this, 90 µl of SO (sesame oil only), KPE-SO, CDE-SO and OTE-SO (plant extracts in oil), were added individually into wells containing bacterial culture. Controls were set up for each set of assays that included uninoculated LB broth (sterility control), and bacterial cultures in LB without the addition of the formulations (untreated control). After 24 h, total biomass (crystal violet assay) and metabolic activity (XTT assay) was measured according to previously published protocols (Kaushik et al., 2016).

### Testing the Effects of Plant-Based Whole Remedies on Biofilm Eradication

To evaluate effects on eradication of pre-formed biofilms, reconstituted traditional remedies were added to 24-h old biofilms of *P. aeruginosa* or *S. aureus*. Overnight cultures of *P. aeruginosa* and *S. aureus* were diluted to a concentration of 10^6^ cells/ml in fresh LB broth, and 100 µl of this diluted culture was added to a 96-well plate such that each well contained 10^5^ cells. The plate was incubated at 37°C under static conditions to allow biofilm formation on the bottom, sides and air-liquid interface of the wells. After 24 h, wells were washed gently (twice) with 150 µl of LB broth to remove any dead or planktonic cells, taking care not to disrupt the biofilm. Following this, 100 µl of SO, KPE-SO, CDE-SO and OTE-SO were added individually to respective wells. Controls were set up for each set of assays that included uninoculated LB broth (sterility control), and pre-formed biofilms treated with LB broth (untreated control). Plates were further incubated at 37°C under static conditions for 24 h. After 24 h, the metabolic activity of the biofilms was measured with the XTT assay; a critical read-out for antimicrobial effects against pre-formed biofilms is the ability to reduce the viable cell count.

### Testing the Effects of Plant-Based “Whole” Remedies Against Planktonic Cultures

We tested the effects of the traditional remedies on planktonic growth of *P. aeruginosa* and *S. aureus* using *in vitro* growth curves. Overnight cultures were diluted to a concentration of 10^7^ cells/ml in fresh LB broth. Each well consisted of 10 µl of this diluted culture of either pathogen (10^5^ cells), with 90 µl of either LB (untreated), SO, KPE-SO, CDE-SO or OTE-SO. Uninoculated LB was used as a sterility control. The plate was incubated at 37°C with shaking in a multimode plate reader (Tecan Infinite 200 PRO) and absorbance was measured at 600 nm (optical density (OD)) every 30 min overnight (∼15–16 h).

## Results and Discussion

### Identifying Potential Anti-biofilm Agents From Historical Treatises

**FIGURE 1:**
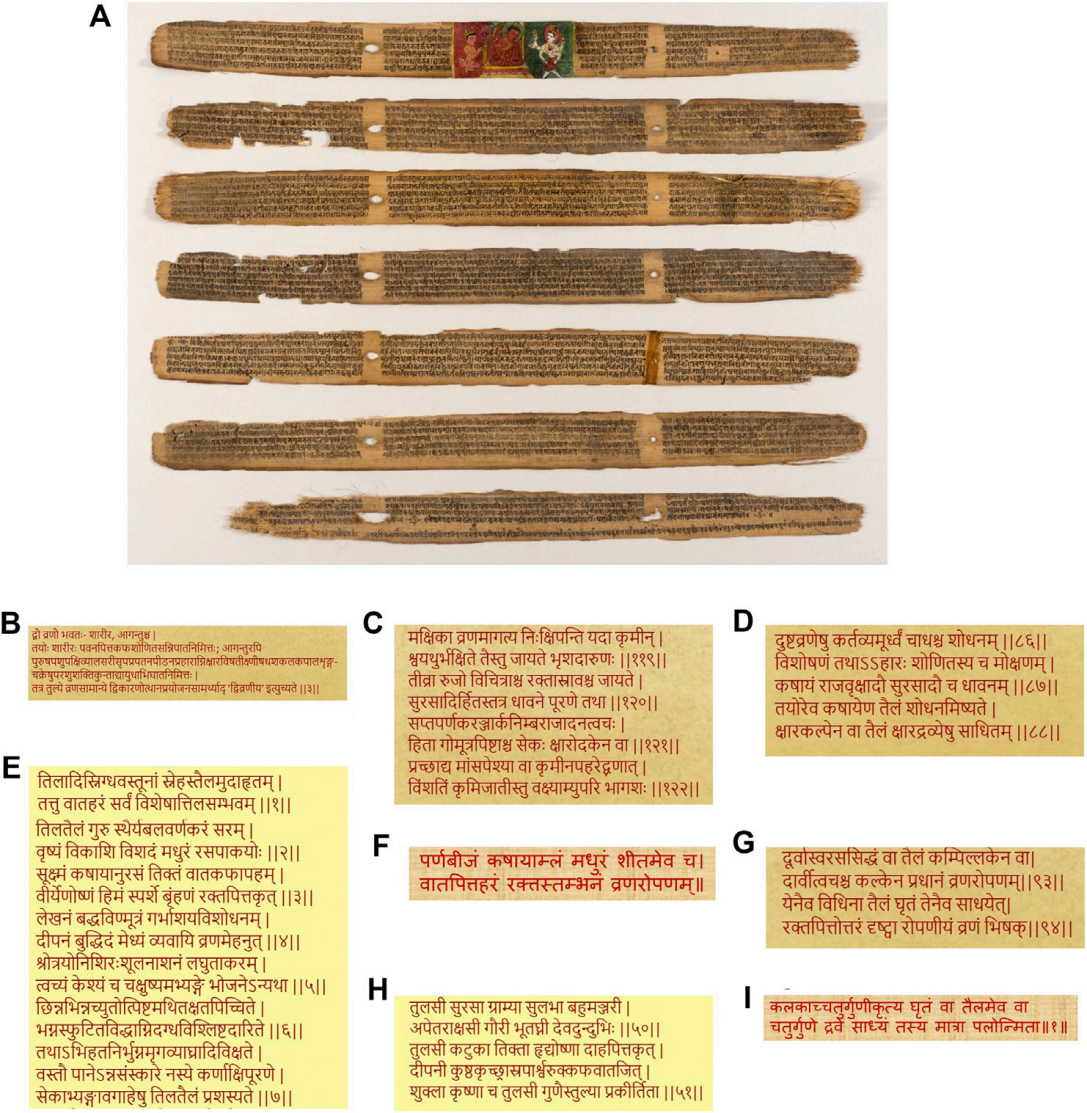
**(A)** *Sushruta-Samhita*, a fundamental text of traditional Indian medicine written on palm leaves. Found in present-day Nepal, the text is dated 12th–13th century, while the art is dated 18th–19th century. Displayed at the Los Angeles County Museum of Art (LACMA). Reproduced in accordance with guidelines (https://www.lacma.org/). Gleanings across historical medical treatises of Ayurveda **(B)** Description of types of wounds in *Sushruta Samhita* **(C)** The problem of infected wounds described in *Sushruta Samhita* as “germination of maggots/worms due to flies on the wound” **(D)** Principles of treatment of infected wounds in *Sushruta Samhita* call for the use of medicinal oils **(E)** Recommendation of the use of sesame oil for medicinal formulations in the Indian Materia Medica or *Bhavprakash Nighantu* **(F)** Use of *Kalanchoe pinnata* or “*Parnabeej*” for wound healing in ‘*Dhravyagun Vignyan* (Ayurvedic text) **(G)** *Cynodon dactylon* or “*Durva*” for wound healing in *Charak Samhita* **(H)** Medicinal roles of *Ocimum tenuiflorum* or “*Tulsi*” in *Bhavprakash Nighantu* **(I)** Preparation practices of plant-based oils with four-fold ratio of components, plant paste, oil and water, from *Sharangdhar Samhita*.

Originating in the Indian subcontinent, Ayurveda is a traditional system of medicine, with historical compendia of practices and preparations built over 2,000 years (Figure 1A). Given the established association of biofilm infections with non-healing, chronic wounds (Bjarnsholt et al., 2008; DeLeon et al., 2014), we searched for traditional antimicrobial remedies with historical applications in the context of wounds. Across the foundational treatises, “*Charaka Samhita*” (compendium by *Charaka*, 1st century AD (e-Samhita - National Institute of Indian Medical Heritage)) and “*Sushruta Samhita*” (compendium by *Sushruta*, 4th century AD (e-Samhita - National Institute of Indian Medical Heritage)) the management of wounds (“*Vrana*”) and chronic, non-healing wounds (“*Dushtavrana*”), have received substantial attention, with evidence of treatment with plant-based remedies (Parasuraman et al., 2014). In the compendium by *Sushruta*, wounds are described to be of two types, “body” wounds (*“Sharir Vran”*), referring to wounds as a consequence of internal disease states, and external wounds (*“Agantu Vran”*) resulting from trauma, burns, and animal bites (Figure 1B; Supplementary Table 1). Descriptions of ailments, and associated remedies, that translated (from Sanskrit) to “wounds” (“*Vrana*”), “wound healing” (“*Vranaropana*”), “ulcers,” and “non-healing ulcers” (“*Dushtavrana*”), were focused on, given their present-day association with biofilm infections (Metcalf and Bowler, 2013; Zhao et al., 2013; Percival et al., 2015; Abdullahi et al., 2016). We found evidence of non-healing wounds in the compendium of *Sushruta*, translated as the “germination of maggots/worms due to flies on the wound can cause pain, swelling and bleeding” (Figure 1C; Supplementary Table 1). It is very likely that in this chronic state of tissue damage or necrosis, bacterial infections in the form of biofilms, will occur and prevail (Bowler et al., 2001). The compendium also describes the principles of management of non-healing wounds (“*Dushtavrana*”) with the practice of washing the wound with a decoction of oil cooked with medicinal herbs, including “*Surasa*” or *“Tulsi*” (Holy Basil) (Figure 1D; Supplementary Table 1). In the Ayurvedic Materia Medica (“*Bhavaprakash Nighantu*” (bhAvaprakAshanighaNTu)), the medical benefits of sesame oil are elucidated in detail, including its recommended use in medicinal oil formulations (Figure 1E; Supplementary Table 1), and sesame oil is widely-used by Ayurvedic practitioners even today. Across compendia, we identified three medicinal plant-based formulations recommended for wound healing (“*Vranaropana”)*, *Kalanchoe pinnata* (“*Parnabeeja*”), *Cynodon dactylon* (Bermuda grass or “*Durva*”) and *Ocimum tenuiflorum* (Holy basil or “*Tulsi*”) (Figures 1F–H; Supplementary Table 1). All three medicinal plants have well known antimicrobial properties (Tatsimo et al., 2012; Cohen, 2014; Marasini et al., 2015).

### Reconstituting Traditional Remedies With Anti-Biofilm Potential in Present-Day

Based on gleanings across compendia, the traditional formulations were prepared by “extracting” ingredients from the medicinal plants into sesame oil. In doing so, we proceeded to reconstitute the “whole” remedy. This is important given that plant-based formulations have been shown to possess varied biofilm activity, depending on whether they are prepared via traditional methods or chemical extractions (Lyles et al., 2017). Further, sesame oil is itself known to possess antimicrobial properties (Nigam et al., 2015; Heidari-Soureshjani, 2016; Lavaee et al., 2019). Remedies of each type of medicinal plant, *Kalanchoe pinnata* (Lam.) Pers. (“*Parnabeeja*”), *Ocimum tenuiflorum* L. (Holy basil or “*Tulsi*”), and *Cynodon dactylon* (L.) Pers. (Bermuda grass or “*Durva*”), were prepared using a mixture of plant paste, distilled water (Alliance Industries, Pune, India) and sesame oil (cold-pressed, Prabhat teel oil, Hanuman Trading Company, India), referred to as KPE-SO, CDE-SO and OTE-SO, respectively. The plants were obtained from a private, cultivated botanical garden in Pune, India (belonging to Dr. Anuradha, Ayurvedic practitioner and author in this work). All medicinal plant names were validated from the Kew Medicinal Plant Names Services. The names of *Ocimum tenuiflorum* and *Cynodon dactylon* were validated as is and the service repository stated non-scientific names as “Holy basil” or “tulsi” for *Ocimum tenuiflorum* and “Bermuda grass” or “durva” for *Cynodon dactylon.* When the Kew Medicinal Plant Names Services was searched for *Kalanchoe pinnata*, it found one plant termed as ‘Kalanchoe pinnata (Lam.) Pers. The preparation practices to reconstitute these plant ingredients in sesame oil were determined from “*Sharangdhar Samhita*” (Figure 1I), which recommends that to prepare decoctions of plant-based medicinal oil, the oil component should be four times that of the plant paste, and the “liquid” component (referring to water or milk) should be four times that of the oil component, resulting in a ratio of 1:16:4 for plant paste, water and oil respectively. It is unclear if this refers to measurement by weight or volume, and we decided to use volume. The recipe also calls for boiling the components together to extract the plant ingredients into the sesame oil. Since the water is allowed to evaporate, the final drug extract ratio (DER), based on four parts of sesame oil for one part of plant paste is 1:4.

### Testing the Effects of the “Whole” Remedies Against Biofilms Using Microtiter-Based Assays and Analysis of Results Based on Minimum Information Guidelines

Infections in chronic, non-healing wounds are most commonly caused by bacterial pathogens *Pseudomonas aeruginosa* and *Staphylococcus aureus* (Serra et al., 2015). We, therefore, evaluated the anti-biofilm effects of the traditional remedies on these two pathogens. The anti-biofilm potential of reconstituted traditional remedies was tested under *in vitro* conditions with microtiter-plate based biofilm assays (Kragh et al., 2019). A mainstay of biofilm studies, these assays are widely-used, rapid, accessible, affordable, relatively easy to perform, and use commonly available materials. Further, a set of minimum information guidelines have been recently published for these assays (Allkja et al., 2020). We evaluated the effects of the remedies on both biofilm formation, and eradication of pre-formed biofilms. We measured total biomass using the crystal violet assay (Kaushik et al., 2016), and metabolic activity of the biofilms using a colorimetric assay with the tetrazolium dye “XTT” (Kaushik et al., 2016). Based on previously published guidelines (Allkja et al., 2020), we have stated our experimental designs and protocols providing details of strains, reagent volumes, and types of microplates. All assays were done with at least two biological replicates (each with three technical replicates) for each strain and for the medicinal formulations. The data was plotted as box and whisker plots. All statistical analysis was performed using GraphPad Prism 8. Data was checked for normality using the Shapiro-Wilk normality test. A one-way ANOVA with Dunnett’s multiple comparison was performed to test for significance. A *p*-value of <0.05 was considered significant. The effects of the remedies were expressed as a percentage reduction compared to the untreated and reported as a range.

### Effects of Traditional Plant-Based Remedies on Biofilm Formation

**FIGURE 2:**
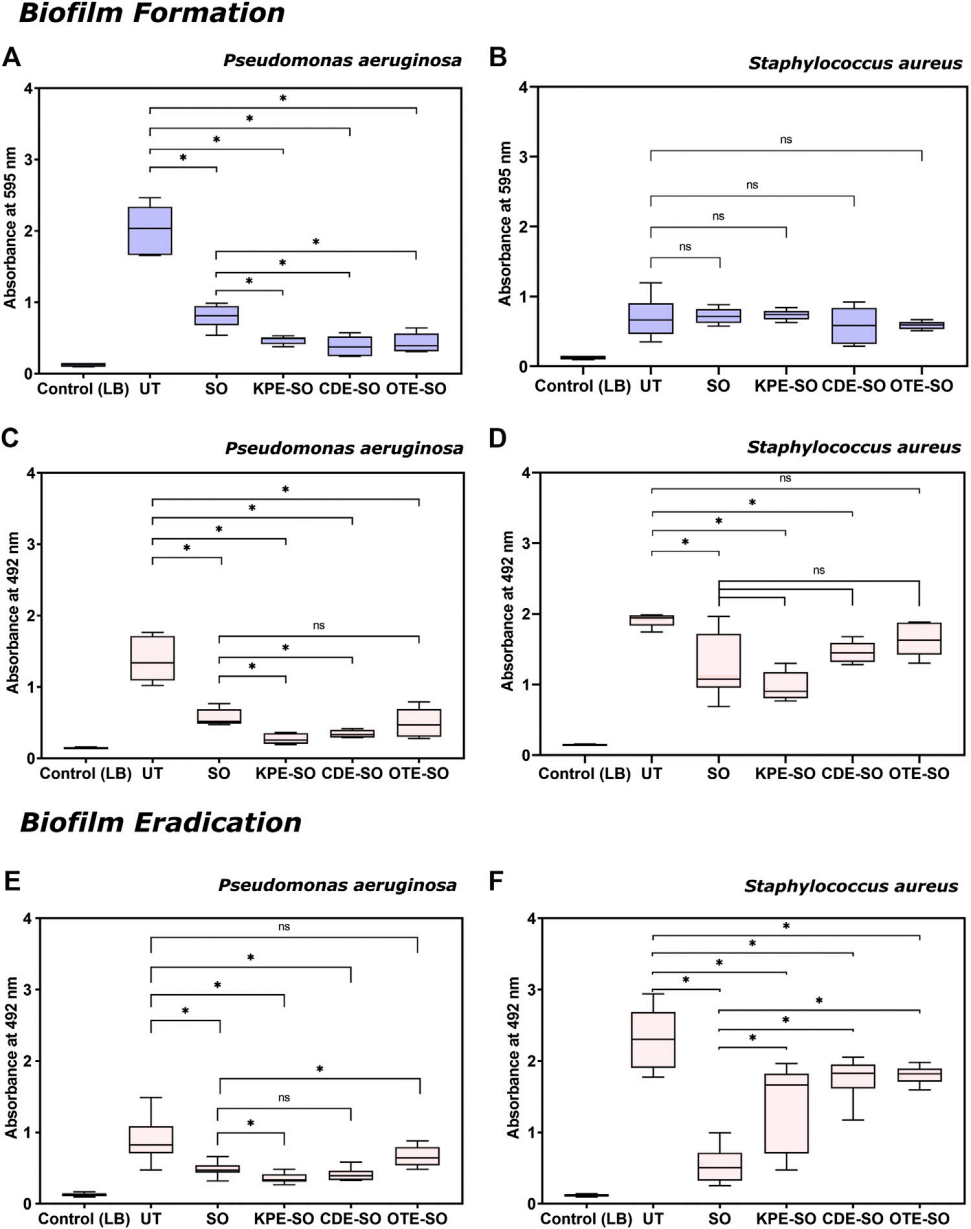
Effects of traditional remedies on the formation of biofilms. **(A,B)** Total biomass quantification (crystal violet assay) and **(C,D)** Quantification of metabolic activity (XTT assay) of *P. aeruginosa* and *S. aureus* biofilms formed in the presence of traditional formulations. Effects of traditional remedies on eradication of pre-formed biofilms (24 h old). **(E,F)** Quantification of metabolic activity (XTT assay) of *P. aeruginosa* and *S. aureus* pre-formed biofilms treated with traditional medicinal formulations (LB-Luria-Bertani medium, UT-untreated biofilm, SO-sesame oil, KPE-SO-*Kalanchoe pinnata*; CDE-SO-*Cynodon dactylon*, OTE-SO-*Ocimum tenuiflorum*). *p value ≤0.05 is considered statistically significant; vs. untreated control, and formulations). At least two biological replicates (each with three technical replicates) were performed for all assays.

As shown in Figure 2A, in the presence of SO, KPE-SO, CDE-SO and OTE-SO, *P. aeruginosa* biofilms showed significantly decreased total biomass, when compared with untreated biofilms (UT). The reduction in the total biomass formed in the presence of sesame oil (SO) and the three traditional remedies was quantified to be 53–66%, 74–80%, 74–88% and 72–85% for KPE-SO, CDE-SO and OTE-SO respectively. This corroborates with results in Figure 2C, that shows a reduction in biofilm metabolic activity, indicating less viable cells (XTT assay), in the presence of SO, KPE-SO, CDE-SO and OTE-SO (48–66%, 75–86%, 71–79% and 49–79% reduction respectively). Our results demonstrate that SO itself has antibiofilm activity. However, the reduction in both biomass and metabolic activity in the presence of KPE-SO and CDE-SO was significant as compared to SO alone, indicating that the plant remedies had an enhanced anti-biofilm effect. For OTE-SO, the enhanced anti-biofilm activity compared to SO was seen only in the case of total biomass, and no significant difference in metabolic activity was observed.

On the other hand, as seen in Figure 2B, none of the three remedies, KPE-SO, CDE-SO and OTE-SO, or sesame oil (SO) alone, showed a significant effect on the biomass formation of *S. aureus* biofilms. However, in Figure 2D, when treated with SO, KPE-SO and CDE-SO there was a significant reduction in the metabolic activity of *S. aureus* biofilms as compared to biofilms grown without treatment (UT), an effect not observed with OTE-SO. Interestingly, there was no significant difference between the KPE-SO and CDE-SO remedies compared to SO alone, indicating that the plant components of the remedies had no further effect on reducing metabolic activity of *S. aureus* biofilms, as compared with sesame oil alone.

### Effects of Traditional Plant-Based Remedies on Biofilm Eradication

As shown in Figure 2E, treatment with SO, KPE-SO and CDE-SO were seen to reduce the viable cell mass of pre-formed 24-h *P. aeruginosa* biofilms quantified as 35–56%, 52–68% and 45–64% reduction respectively (as compared with UT). Of the three remedies, only KPE-SO showed an increased reduction in viable cells as compared with SO alone. Infact, OTE-SO treatment was observed to show a marginal increase in cell viability (as compared with SO), an effect that could possibly be attributed to select nutrients provided by the plant-based ingredients or antagonistic interactions between components. When pre-formed *S. aureus* biofilms were treated with SO, KPE-SO, CDE-SO and OTE-SO for 24 h (Figure 2F), a significant reduction in the viability of biofilms (as compared with UT) was seen with SO (66–87%) and KPE-SO (19–68%), and a lesser effect was observed with CDE-SO and OTE-SO (13–35% and 17–27% reduction respectively). However, when compared with SO alone, treatment with all three plant-based remedies (in sesame oil) for 24 h showed higher cell viability in biofilms, possibly indicating interactions between plant-based components and sesame oil.

### Testing the Effects of the “Whole” Remedies Against Planktonic Cultures

**FIGURE 3:**
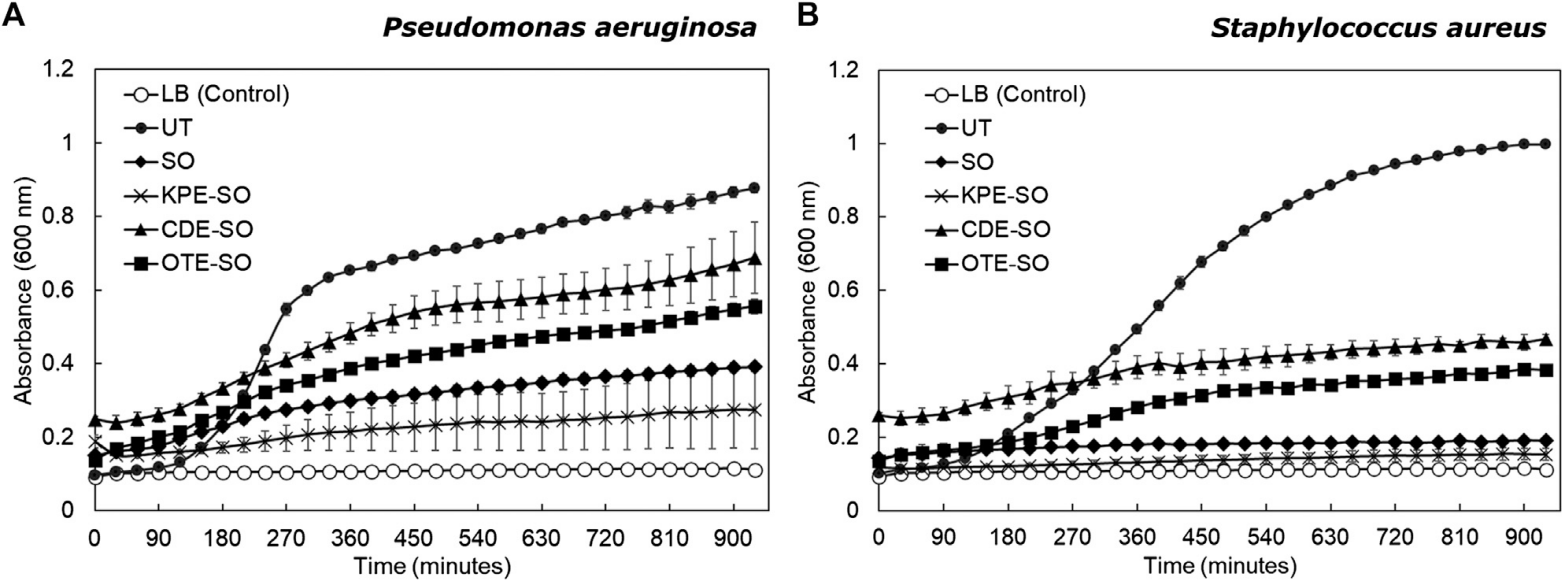
Effects of traditional remedies on planktonic growth of *P. aeruginosa* and *S. aureus*. **(A)** *P. aeruginosa* and **(B)** *S. aureus* were grown in the presence of LB (UT), sesame oil (SO), or the three plant-based formulations (KPE-SO, CDE-SO, and OTE-SO). Two biological replicates (each with three technical replicates) were performed.

As seen in Figure 3, for both pathogens, sesame oil and the three plant-based formulations were observed to inhibit planktonic growth compared to the untreated culture (grown in LB). *P. aeruginosa* untreated planktonic cultures showed a doubling time of 29.3 ± 4.0 min (mean ± standard deviation). *P. aeruginosa* planktonic cultures treated with CDE-SO and OTE-SO showed inhibited growth as compared with untreated cultures (doubling times 65.4 ± 16.5 min and 113.5 ± 28.0 min respectively); SO and KPE-SO treated cultures showed almost no growth. A similar trend was seen for *S. aureus* (Figure 3B). Untreated planktonic *S. aureus* cultures showed a doubling time of 40.8 ± 1.7 min. *S. aureus* planktonic growth was inhibited in the presence of the plant-based formulations, with KPE-SO and sesame oil treated cultures showing no growth at all. As stated previously, observations that planktonic cultures treated with SO show a greater inhibitory effect, as compared to CDE-SO and OTE-SO, could be attributed to select nutrients provided by the plant-based ingredients or antagonistic interactions between components; effects that could be explored further. Interestingly, comparison of biofilm (Figure 2) and planktonic (Figure 3) growth states reveals that the plant-based reemdies had differential effects on these two modes of growth. For example, treatment with CDE-SO and OTE-SO showed greater inhibition of total biomass formation in comparison with SO (Figure 2A), however under planktonic conditions, SO was more inhibitory (Figure 3A). CDE-SO also resulted in reduced metabolic activity during biofilm formation as compared with SO (Figure 2C). For *S. aureus*, while SO and the plant-based formulations inhibited planktonic growth (Figure 3B), they had no significant effects (as compared with UT) on total biomass during biofilm formation (Figure 2B); an inhibitory effect on metabolic activity was observed (Figure 2D).

### Summary of Findings

Based on the results, the plant-based “whole” remedies display differential effects on biofilm formation and eradication of preformed biofilms for the two biofilm-forming wound pathogens. In the presence of sesame oil, and plant remedies KPE-SO, CDE-SO and OTE-SO, *P. aeruginosa* biofilms showed significantly decreased total biomass and metabolic activity, when compared with untreated biofilms. On the other hand, none of the three remedies, KPE-SO, CDE-SO and OTE-SO, or sesame oil alone, showed a significant effect on the biomass formation of *S. aureus.* When treated with SO, KPE-SO and CDE-SO there was a significant reduction in the metabolic activity of *S. aureus* biofilms as compared to biofilms grown without treatment. Importantly, sesame oil alone is observed to have anti-biofilm effects.

Treatment with sesame oil, KPE-SO and CDE-SO were seen to reduce the metabolic activity of pre-formed *P. aeruginosa* biofilms, however only KPE-SO showed an increased reduction in viable cells as compared with sesame oil alone. Similarly, a significant reduction in the viability of pre-formed *S. aureus* biofilms was seen with sesame oil, KPE-SO, CDE-SO and OTE-SO.

The differences observed with sesame oil and the plant-based remedies on the biofilm and planktonic state of *P. aeruginosa* and *S. aureus*, reiterate the need to move beyond standard antimicrobial testing practices which typically test against planktonic states, and include anti-biofilm testing as part of evaluation of traditional formulations.

### Considerations and Challenges in the “Treatise to Test” Pipeline for “Ancientbiotics”

**FIGURE 4:**
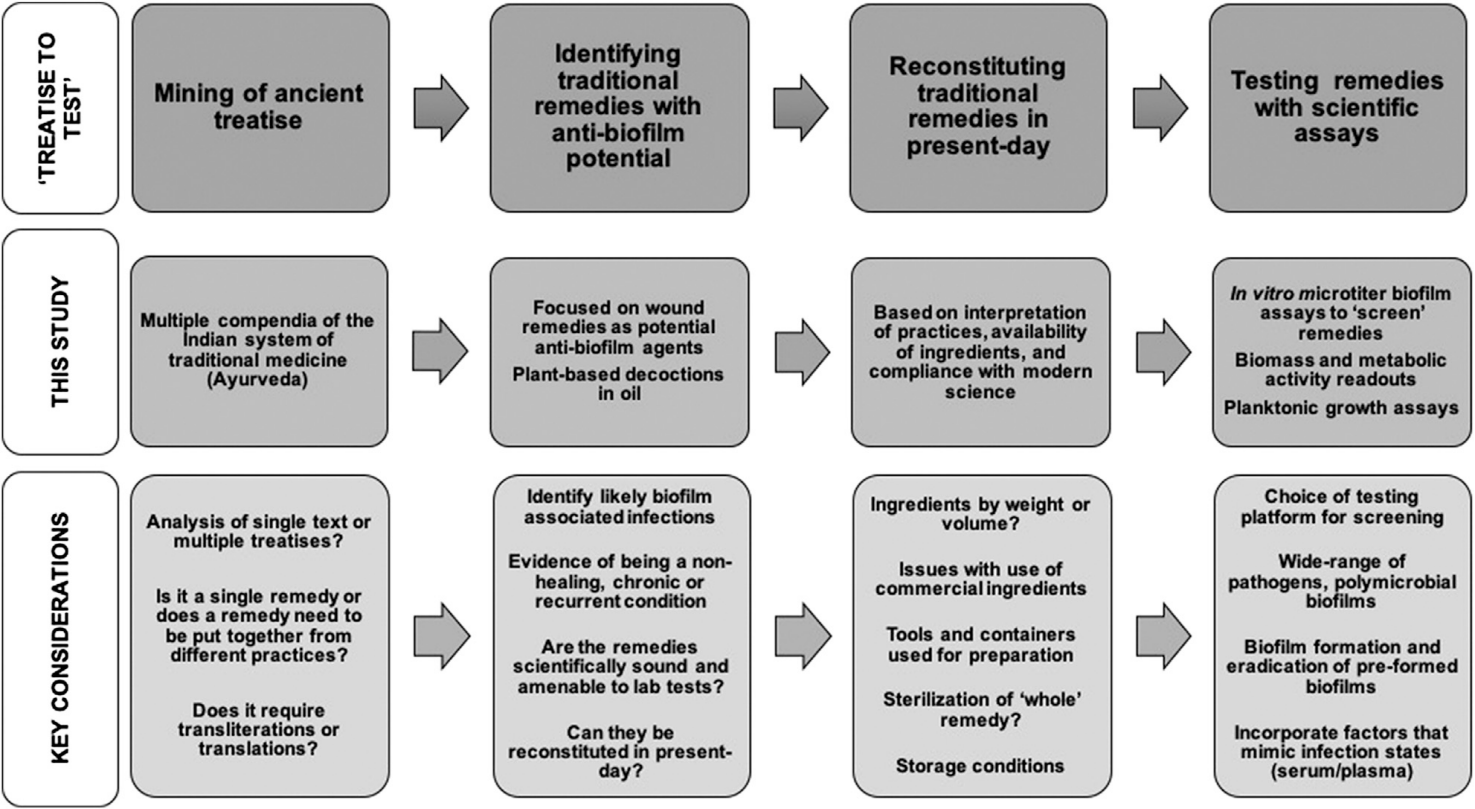
Common themes and key considerations in the “treatise to test” phase of developing as anti-biofilm approaches.

We have spanned medical and technical knowledge from the pre-2nd century CE to the present-day to evaluate the anti-biofilm effects of traditional formulations using contemporary scientific assays and analysis. Based on our approach and results, we shed light on the considerations and challenges relevant to evaluating traditional medicinal remedies as anti-biofilm agents, focusing on the “treatise to test” phase of the development pipeline (Figure 4).

### Identifying traditional remedies with anti-biofilm potential from historical treatises

Ancient texts will not explicitly include the term “biofilm” (or a translated term), therefore the context of medicinal applications in the treatise was used for insights into the infection state. Given that microbial biofilms are widely implicated in non-healing wounds (Percival et al., 2015), we focused on identifying traditional wound remedies that could be explored as potential anti-biofilm agents. On similar lines, other biofilm infection states, such as eye (blepharitis or stye (Bispo et al., 2015)) or ear infections (chronic suppurative otitis media (Lee et al., 2009)), could also be explored.

### Evidence of Remedies Being Scientifically Sound and Testable in the Laboratory

Based on historical findings, we reconstituted three traditional plant-based remedies in sesame oil, *Kalanchoe pinnata* or *“Parnabeeja,” Ocimum tenuiflorum* or *“Tulsi,” Cynodon dactylon* or *“Durva.”* Sesame seed oil (*Sesamum indicum*) is known to possess antimicrobial properties (Nigam et al., 2015; Heidari-Soureshjani, 2016; Lavaee et al., 2019), and is widely used as an “oil pulling” agent to combat dental plaque formation (Anand et al., 2008; Thaweboon et al., 2011; Naseem et al., 2017; Shanbhag, 2017). In an *in vitro* model of oral infection with saliva-coated microtiter plates, sesame oil displayed antibacterial activity against *S. mutans* biofilms. These effects could explain the use of sesame oil in ancient medicinal formulations. *Kalanchoe pinnata* (Akinpelu, 2000; Akinsulire et al., 2007; Gurnani et al., 2014; Kerekes et al., 2019)*, Cynodon dactylon* (Rao et al., 2011; Abdullah et al., 2013; Chinthala et al., 2017), and *Ocimum tenuiflorum* (Cohen, 2014; Yamani et al., 2016), possess well-known medicinal properties, and are used for a variety of ailments in traditional medicine. Leaf extracts of these plants (methanol or ethanol-based) have been reported to have *in vitro* antimicrobial effects against *P. aeruginosa* and *S. aureus,* using disc-diffusion assays (Akinpelu, 2000; Agarwal et al., 2010; Rao et al., 2011; Yamani et al., 2016; Obioma et al., 2017)*.* Therefore, these three plant-based oil formulations were determined to be scientifically sound and testable. This is important to consider as other proposed treatments for infected wounds in treatises, such as dietary modifications, emetics and purgatives, cannot be tested in the laboratory and are out of the scope of our work (Heinrich et al., 2020).

### Reconstituting Traditional Remedies in Present-Day

To reconstitute the plant-based remedies in sesame oil, we followed preparation practices as faithfully as possible, however, certain modifications were necessary based on the availability of materials, and to ensure compliance with modern scientific practices. Based on a historical reference*,* the plant paste, water and oil were recommended to be used in a ratio of 1:16:4, however, it is unclear if this refers to weight or volume. The unit of measurement in traditional Indian medicine is typically described as “weight of a seed” or “enough to fill your hand or palm,” the latter possibly indicating volume. We used volume to measure all components. This is an important initial consideration, and one way to overcome the lack of clarity could be to prepare different versions of the remedy.

### Use of Commercial Ingredients

The recipes call for “*teel oil*,*”* or sesame seed oil, which in ancient times is likely to have “been made from scratch,” just as the plant pastes. We did not have access to the equipment or familiarity with the processes needed to “press” oil from sesame seeds, and hence used sesame oil procured from a local source. This commercial oil source is routinely used by present-day Ayurvedic practitioners. It would be important to consider variations in the properties of the oil based on the source of the raw seeds and processing conditions (Shahidi et al., 1997).

### Preparation of Variations of the Formulations

The recipes also did not call for any specific type of metal container for remedy preparation, and we used a stainless steel pot. Leaching of trace amounts of metals (silver, aluminum, copper) from containers could contribute to the effects of formulations (Turner, 2017). We also “modernized” the preparation practices to use a mechanized grinder (as opposed to physical grinding with rock or stone), to ensure we obtained a uniform, consistent paste. This could influence the “release” of ingredients into the plant paste, and use of a mortar and pestle could be an intermediate option. Similarly, the liquid component of the remedy recommends the use of water or milk, and we decided to use water. One way to account for these factors would be to prepare and test multiple variations of the traditional formulations.

### Sterilization of the Formulations

Notably, the recipe did not call for any specific sterilization practices. This could be because the prolonged boiling process may itself have a sterilizing effect, or that microbes, if any, would be part of the “remedy.” It is important to consider the effects that modern-day sterilization practices (high temperature, filtration) might have on the constituents of the formulation. Accordingly, we did not sterilize the remedies, however, when incubated as controls, uninoculated sesame oil and plant-based formulations did not show presence of microbial growth (data not shown).

### Long Term Storage

We prepared and tested all the three formulations in a single batch. Sesame oil has high oxidative stability (Hashempour-Baltork et al., 2018), and can be stored at room temperature for long periods of time. However, it would be important to consider possible deterioration in antimicrobial activity of the formulation, depending on the ingredients and their properties, and testing could be done in intervals of time.

### Testing the Effects of Traditional Remedies With Scientific Assays and Analyses

To test the anti-biofilm potential of reconstituted remedies we used standard microtiter-plate based biofilm assays. These are *in vitro* laboratory-based assays, in which thin biofilms form on the bottom and sides of polystyrene wells (Kragh et al., 2019). Inspite of these obvious limiting factors, we chose to test the remedies with these assays for several reasons. Microtiter based biofilm assays can be used to rapidly test a large number of compounds and enable semi-quantitative estimations of biomass and metabolic activity of biofilms (Kragh et al., 2019). In addition, a recent set of technical guidelines have been published to enable consistent testing and reporting (Allkja et al., 2020). Therefore, these widely-employed assays lend well as “screening” platforms. While these assays have been used to test various natural antimicrobial compounds, they are usually single ingredients and chemical extracts; here we show that these assays can be used to test “whole” traditional remedies. It is important to consider that the medicinal preparations are in a form suitable for testing with these assays, for example, liquid preparations as opposed to a gel or cream. With these assays, the reconstituted remedies displayed differential activity on biomass formation and metabolic activity, against biofilm and planktonic states of *P. aeruginosa*, a Gram negative pathogen, and *S. aureus*, which is Gram positive. This highlights the value of these assays as an initial screening tool for traditional remedies. Notably, these effects were seen against planktonic cultures, pre-formed biofilms as well as on the formation of new biofilms. This underscores the importance of testing a wide range of pathogens, and planktonic vs. different biofilm states.

### Clinically-Relevant Testing and Further Evaluation

We have reconstituted and tested plant-based whole formulations as described in historical documents. The recommended recipe details a specific ratio of 1:16:4 for the plant paste, water, and sesame oil, which we suitably recapitulated. In the traditional context in which they were used, the remedies, prepared as per the recommended formulation and concentrations, were applied to the surfaces of wounds. We have recapitulated this “dosage” by applying the whole remedy to the surface of the biofilm (eradication assays) or studied the growth of biofilms in this formulation (biofilm formation assays) (Heinrich et al., 2020).

Typically, ethnopharmacological studies have focused on isolating and testing the antimicrobial effects of single compounds (Cowan, 1999; Eloff, 2019). However, reconstituted plant remedies possess a diverse range of ingredients, and it is increasingly being recognized that these combined effects contribute to the effects of the “whole remedy” (Cowan, 1999; Khameneh et al., 2019). The remedies reconstituted and tested in this work are complex plant formulations, and as part of the next steps, it would be important to identify and investigate interactions between the biologically active compounds in the formulations. For this, approaches such as mass spectrometry-based metabolomics that are capable of identifying biologically active compounds in plant extracts (Caesar et al., 2019a; Caesar et al., 2019b) can be applied.

The biofilm inhibitory effects of the medicinal formulations could be attributed to the properties of ingredients, that include specific antimicrobial effects or interactions with the biofilm matrix (Akinpelu, 2000; Akinsulire et al., 2007; Abdullah et al., 2013; Yamani et al., 2016). On the other hand, the possibility of a physical effect of the oil-based remedies, that prevents bacterial attachment or reduces the availability of oxygen is also possible (Kerekes et al., 2019). One study that employed crystal violet-based microtiter assays to evaluate the effects of essential oils (thyme, cinnamon, marjoram) on biofilm formation allowed the bacteria to attach to the substrate for 4 h, after which the oils were added (Kerekes et al., 2019). The addition of certain oils resulted in a decrease in the number of attached cells, leaving mainly damaged cells (observed under SEM). Therefore, after an initial screening, the selected promising remedies can be further examined for the nature of their activity (bactericidal, bacteriostatic or matrix-modifying), and possible cytotoxicity, with *in vitro* systems that mimic infection sites and animal models (Harrison et al., 2015; Furner-Pardoe et al., 2020). Though biological model systems are more clinically-relevant, they are not high-throughput, and require substantial expertise, making them unsuitable for preliminary testing.

### Historical and Philosophical Aspects

Studying traditional remedies requires open-minded teams, which include trained practitioners of ancient medical systems. Our team consisted of a doctor of modern medicine, a traditional medical practitioner, and scientists. This combined expertize made it possible to mine ancient treatises written in Sanskrit, translate them into English, and reconstitute, test and analyze them with modern scientific practice. It was certainly helpful that Ayurveda is still practiced and taught across India, and practitioners are familiar with these treatises and preparations. Traditional medicine is widely practiced in low-resource and underserved parts of the world (Mulaudzi et al., 2017; Lu et al., 2019), it is important that researchers from these regions are included, and tests and guidelines proposed are accessible to them.

Developing historical remedies as anti-biofilm agents holds enormous potential, and there are a plethora of traditional remedies to be explored (Shah, 2011; Bhattacharya, 2012; Lau and Plotkin, 2013; Lu et al., 2019). It is evident that there are several considerations and challenges involved in bringing these remedies out of treatises and testing their effects in the laboratory. While the exact details may vary across systems of medicine, historical texts, and preparation practices, this “treatise to test” phase will include certain common themes. We believe that this study will serve as a starting point for future investigations along these lines.

## Data Availability Statement

The datasets presented in this study can be found in online repositories. The names of the repository/repositories and accession number(s) can be found in the article/supplementary material.

## Ethics Statement

No human studies are presented in this manuscript. No animal studies are presented in this manuscript. No potentially identifiable human images or data is presented in this study.

## Author Contributions

VM, SK, AB, and KSK conceived and designed the study. AB and KSK identified the traditional medicinal remedies. AB prepared the medicinal remedies. VM and SK did the experimental assays and statistical analyses. VM, SK, and KSK analyzed the data and wrote the manuscript. SK did the additional experiments based on reviewers’ recommendations, and SK and KSK revised the manuscript.

## Funding

This work was funded by the Ramalingaswami Re-entry Fellowship, Department of Biotechnology, Government of India (BT/HRD/35/02/2006, to KSK).

## Conflict of Interest

The authors declare that the research was conducted in the absence of any commercial or financial relationships that could be constructed as a potential conflict of interest.
